# Transoral Robotic Lingual Tonsillectomy for Pediatric Obstructive Sleep Apnea

**DOI:** 10.1002/lary.70267

**Published:** 2025-11-26

**Authors:** Daniel J. Campbell, Daron Excel, W. Jack Palmer, Heather C. Nardone, Douglas R. Johnston

**Affiliations:** ^1^ Department of Otolaryngology Sidney Kimmel Medical College at Thomas Jefferson University Philadelphia Pennsylvania USA; ^2^ Department of Otolaryngology Northwestern University Feinberg School of Medicine Chicago Illinois USA; ^3^ Division of Pediatric Otolaryngology Nemours Childrens Health Delaware Wilmington Delaware USA; ^4^ Division of Pediatric Otolaryngology Ann & Robert H. Lurie Children's Hospital of Chicago Chicago Illinois USA

**Keywords:** lingual tonsillectomy, obstructive sleep apnea, pediatric

## Abstract

**Objectives:**

Our objective was to investigate the safety and efficacy of transoral robotic surgery (TORS) lingual tonsillectomy (LT) with and without epiglottopexy and to compare it with non‐robotic approaches for children with obstructive sleep apnea (OSA).

**Methods:**

A multi‐institutional retrospective chart review was conducted on 56 patients who underwent TORS LT (32 males, 24 females; mean age 11.7 ± 4.2 years; 29 syndromic) between 2015 and 2024 at 2 tertiary care pediatric hospitals. Preoperative and postoperative polysomnogram data were collected and analyzed, along with clinical and demographic data to analyze predictors of surgical success.

**Results:**

Of the patients with pre‐ and postoperative polysomnogram, 26 patients (60.5%) were considered a surgical success. Significant improvements were seen in apnea hypopnea index (mean reduction of −14.39 ± 5.2; *p* = 0.0079), apnea hypopnea index during rapid eye movement (mean reduction of −25.89 ± 8.8; *p* = 0.0057), and O2 nadir (mean improvement of 5.73% ± 1.64%; *p* = 0.0012). There were no significant differences in the proportion of surgical success based on preoperative OSA severity, presence of a syndrome, or presence of obesity. Eleven patients experienced complications (19.6%), including postoperative hemorrhage requiring operative intervention (8.9%) and oropharyngeal scarring (3.6%).

**Conclusion:**

For pediatric patients with OSA and lingual tonsillar hypertrophy, TORS is an effective and safe method of performing LT, with benefits including excellent 3‐dimensional visualization and a greater ability to distinguish the lingual tonsil/tongue musculature interface. While this is the largest pediatric TORS LT study to date, further research with larger sample sizes and additional sleep and quality of life metrics would add to the existing literature.

**Level of Evidence:**

4.

## 1 Introduction

Otolaryngologists began to explore the feasibility of transoral robotic surgery (TORS) as an approach to adult head and neck cancers in 2005 when Hockstein et al. published a study using the da Vinci surgical robot on an airway mannequin [1]. Soon after, the first TORS procedures were performed on adults for supraglottic and base of tongue neoplasms [2]. In 2007, the first study exploring the safety and applicability of TORS in pediatric populations was published, which helped pave the way for broadening indications for this technology [3]. Since then, multiple studies have been published using TORS in pediatric populations for excision of benign oropharyngeal and laryngeal pathologies [4] and laryngeal cleft repairs [5]. Notably, in the largest TORS case series to date with 48 total patients, Zdanski et al. found that robotic type 1 laryngeal cleft repair had better postoperative swallow outcomes compared with traditional repairs. Several studies have been published on TORS lingual tonsillectomy (LT), likely the most common indication for pediatric TORS [6, 7].

Lingual tonsillar hypertrophy is a frequent contributor to obstructive sleep apnea (OSA), especially in children who have already undergone tonsillectomy and/or adenoidectomy and have residual OSA. Previous research has demonstrated that pediatric LT is a safe and effective approach to relieving OSA, with success rates ranging from 50% to 80%, although a paucity of postoperative polysomnographic data makes validation of surgical success challenging [6, 8, 9, 10, 11]. Many children with Down syndrome (DS) have residual OSA, the causes of which are multifactorial. Moreover, an increased percentage of children with DS have lingual tonsillar hypertrophy; accordingly, children with DS comprise a sizeable cohort of pediatric LT studies [6, 8, 9]. Additionally, lingual tonsillar hypertrophy is also relatively more common in patients with obesity [6, 8, 9, 11, 12] and moderate‐to‐severe preoperative OSA (apnea hypopnea index [AHI] ≥ 5) [12, 13].

Several LT techniques under microscopic or endoscopic visualization have been used, including monopolar electrocautery [9], coblation [13], and microdebrider dissection [14]. While endoscopic and microscopic techniques have shown to be effective at alleviating airway obstruction [8, 9, 10, 13, 14, 15], there are some advantages to robotic surgery, namely 3‐dimensional visualization, small robotic arms allowing improved access, and precise movements with wristed robotic instrumentation [16, 17, 18, 19]. However, potential downsides of the robotic platform are accessibility to TORS training, high robot purchase price, and a lack of haptic feedback in the majority of systems in use today [20].

Limitations of TORS LT studies include small sample sizes and lack of comparison to endoscopic approaches reported in the literature [21, 22]. There are two principal aims of this study: first, to explore multi‐institutional outcomes of TORS LT in children with OSA with enhanced patient numbers and second, to compare TORS LT with standard techniques using outcomes defined in the literature.

## 2 Methods

After approval by the Lurie and Nemours Children's Hospital Institutional Review Boards, a retrospective chart review was conducted on all patients who underwent TORS LT with or without epiglottopexy for OSA between 2015 and 2024 at two free‐standing tertiary care pediatric hospitals. All patients underwent either preoperative sleep state endoscopy or office‐based laryngoscopy to determine the presence of lingual tonsillar hypertrophy. All LT procedures during the study period at both institutions by the senior authors were performed using the TORS technique. The attending surgeon, or a supervised fellow where clinically appropriate, performed all surgeries and ensured adequate lingual tonsil tissue removal prior to the completion of the procedure. Of note, TORS is also used in a large minority of patients for oropharyngeal mass and vallecular cyst removal between both included institutions. At publication, senior authors D.J. and H.N. have completed over 50 and 30 TORS procedures, respectively. Patients received non‐narcotic pain control (ibuprofen and acetaminophen) postoperatively Every attempt was made to schedule patients for a postoperative follow‐up at 1 month, with additional follow‐up as indicated for new symptoms or concerns.

Demographic and clinical data including gender, age, preoperative body mass index (BMI), presence of a syndrome, and prior surgical procedures were collected. When available, pre‐ and postoperative polysomnogram findings were recorded, including AHI, AHI during rapid eye movement (REM AHI), and O2 nadir. Surgical and hospital course information such as presence of a concurrent procedure, procedure length, and length of stay were collected. Concurrent procedures were defined as an additional procedure other than epiglottopexy in the same surgical episode. Epiglottopexy was performed at the discretion of the surgeon based on the degree of epiglottic retroflexion. Procedure length was defined as the time between the start and end of the procedure. Postoperative complications were documented, including postoperative hemorrhage, new‐onset dysphagia, oropharyngeal scarring, and readmission for pain, dehydration, and/or poor oral intake. New‐onset dysphagia was defined as documented difficulty with oral intake with or without additional means of enteral access persisting beyond 30 days postoperatively. Complications were identified through the review of the inpatient stay records, postoperative follow‐up visits, and telephone encounters. Definitions of complications were identical across both study sites.

OSA severity was defined using standard pediatric AHI stratifications: mild (1 to 4.9), moderate (5 to 9.9), and severe (10 or greater). Surgical success was defined as postoperative AHI < 5 or ≥ 50% reduction in AHI from preoperative baseline. These criteria were adapted from prior literature on surgical success in pediatric OSA [12, 21].

All statistical analyses were conducted with SPSS Statistics, version 28.0.1.0 (IBM Corp., Armonk, NY). Demographic calculations were made using descriptive analyses. All statistical analyses were performed with a significance threshold of *p* < 0.05. Differences in the mean procedure length between surgeries with and without a concurrent procedure were compared using a two‐tailed, non‐paired *t*‐test. Pre‐ and postoperative sleep study data were compared using two‐tailed, paired *t*‐tests. Differences in surgical success frequency were compared using separate chi‐squared or Fisher's exact tests for each independent variable: OSA severity class, sex, and presence of syndrome or preoperative obesity. Pre‐ to postoperative changes in AHI were compared using separate non‐paired, two‐tailed *t*‐tests for each of the same independent variables listed above. Mean difference was defined as postoperative value minus preoperative value.

Univariate logistic regression analyses were performed to assess associations between each independent variable and the binary dependent variable of surgical success. Independent variables included age (in years), gender, presence of obesity, preoperative BMI, presence of a syndrome, presence of severe OSA, preoperative AHI, and preoperative O2 nadir. Multivariate linear and logistic regression analyses were performed to identify independent predictors of surgical success (binary variable) and delta AHI (continuous variable), respectively. When appropriate, quantitative independent variables (i.e., preoperative AHI) were used instead of qualitative independent variables (i.e., OSA severity class) for powering purposes.

## 3 Results

A total of 56 patients were included for analysis. Mean age was 11.7 (±4.2) years (Table 1). Thirty‐two (57.1%) were male, and 24 (42.9%) were female. Mean preoperative BMI percentile was 78.7 (±28.0), and 34 patients (60.7%) were considered obese. The 29 (51.8% of cohort) syndromic patients were further classified as Trisomy 21 (27 patients; 93.1%), Schaaf‐Yang (1 patient; 3.4%), and Prader‐Willi (1 patient; 3.4%). Forty‐nine patients (87.5%) had undergone prior surgical procedures to treat OSA. Prior surgical procedures included adenotonsillectomy (45 patients, 80.4%), tonsillectomy only (2 patients, 3.6%), supraglottoplasty (2 patients, 3.6%), and tracheostomy (2 patients, 3.6%).

**TABLE 1:** Demographic data.

Characteristic	Number (% total)
Gender (% total)	
Male	32 (57.1)
Female	24 (42.9)
Age (±SD)	
Months	144.3 (50.2)
Years	11.7 (4.2)
Preoperative BMI percentile (±SD)	78.7 (28.0)
Obese (% total)	
Yes	34 (60.7)
No	22 (39.3)
Syndromic	
Yes	29 (51.8)
No	27 (48.2)
Prior surgical procedures	
Yes	49 (87.5)
No	7 (12.5)
Preoperative OSA severity	
Mild	3 (5.4)
Moderate	8 (14.3)
Severe	42 (75.0)

*Note*: Obese is defined as BMI ≥ 95th percentile. Preoperative OSA severity includes all patients with preoperative sleep study data with or without a postoperative sleep study.

Abbreviations: BMI, body mass index; OSA, obstructive sleep apnea; SD, standard deviation.

Of the 56 included patients, 16 (28.6%) underwent concurrent epiglottopexy and 15 (26.8%) underwent an additional concurrent procedure. Concurrent procedures included revision adenoidectomy (7 patients, 12.5%), turbinate reduction (3 patients, 5.4%), revision tonsillectomy (3 patients, 5.4%), ear tube placement (1 patient, 1.8%), and otologic exam under anesthesia (1 patient, 1.8%). Mean procedure length for TORS LT only was 71.22 ± 28.4 min compared with 109.93 ± 39.0 min for TORS LT with a concurrent procedure (*p* < 0.001). No procedures required conversion to a traditional technique with suspension laryngoscopy. Mean length of stay was 1.46 ± 1.2 days. Mean time from surgery to the most recently documented follow‐up was 38.8 ± 29.1 months.

Of the 56 included patients, 43 (76.8%) had both pre‐ and postoperative polysomnograms. Median time from surgery to postoperative polysomnogram was 108 days (range: 41 to 336 days). Of the 43 patients with both pre‐ and postoperative polysomnograms, 26 (60.5%) were considered a surgical success, while 17 (39.5%) were not. Of the 26 patients who were considered a surgical success, 2 (7.7%) had postoperative AHI < 5 only, 16 (61.5%) had ≥ 50% reduction from preoperative AHI only, and 8 (30.8%) met both criteria. Of the 17 patients who were not considered a surgical success, 9 (52.9%) had improvement in postoperative AHI. Significant improvements were seen in AHI (mean difference of −14.39 ± 5.2; *p* = 0.0079), REM AHI (mean difference of −25.89 ± 8.8; *p* = 0.0057), and O2 nadir (mean difference of 5.73% ± 1.64%; *p* = 0.0012) (Table 2). The mean AHI reduction percentage per patient was 26.8% (±83.5). Twenty (46.5%) of the patients experienced a shift to a lower OSA severity class (Table 3). Additional stratifications based on increasingly lower AHI cuts (e.g., AHI < 3, AHI < 1) are listed in Table 3.

**TABLE 2:** Preoperative versus postoperative sleep study metrics.

Parameter	Mean preop (±SD)	Mean postop (±SD)	*N*	Mean difference (±SE)	*p*
AHI	34.91 (30.32)	20.52 (26.28)	43	−14.39 (5.159)	0.0079
REM AHI	53.72 (36.64)	27.83 (30.86)	35	−25.89 (8.774)	0.0057
O2 nadir	81.33%	87.05%	40	+5.73% (1.64%)	0.0012

*Note*: Mean difference is defined as postoperative value minus preoperative value. Statistics performed using a paired, 2‐sided *t*‐test with significance set as alpha = 0.05. This comparison only includes patients with both pre‐ and postoperative sleep study data for a given variable. If a patient did not have both a pre‐ and postoperative data point, they were excluded from analysis.

Abbreviations: AHI, apnea hypopnea index; postop, postoperative; preop, preoperative; REM AHI, apnea hypopnea index during rapid eye movement; SD, standard deviation; SE, standard error.

**TABLE 3:** Sensitivity analysis using alternative perioperative AHI criteria.

Criterion definition	Met criteria (% total)	Did not meet criteria (% total)
Any reduction in AHI	35 (81.4)	8 (18.6)
Reduction in AHI ≥ 50%	24 (55.8)	19 (44.2)
Postoperative AHI < 5	10 (23.3)	33 (76.7)
Postoperative AHI < 3	7 (16.3)	36 (83.7)
Postoperative AHI < 1	2 (4.7)	41 (95.3)
Reduction in AHI ≥ 50% and postoperative AHI < 5	8 (18.6)	35 (81.4)
Reduction in AHI ≥ 50% or postoperative AHI < 5	26 (60.5)	17 (39.5)
Shift to lower OSA severity class	20 (46.5)	23 (53.5)

Abbreviations: AHI, apnea‐hypopnea index; OSA, obstructive sleep apnea.

There were no significant differences in the proportion of surgical success based on preoperative OSA severity, presence of a syndrome, presence of obesity, or presence of a concurrent procedure (Table 4). Female patients had a significantly higher proportion of surgical success compared with that of male patients (*p* = 0.002). Female patients did not have a higher proportion of severe preoperative OSA compared with male patients (*χ* ^2^ = 0.074, *p* = 0.786), nor did they have a significantly higher preoperative AHI (mean difference = 5.29 ± 8.8, *p* = 0.550). There were no significant differences in delta AHI based on OSA severity, presence of a syndrome, obesity, gender, or presence of a concurrent procedure (Table SI). An additional analysis was performed to determine if the complication rate differed significantly with concurrent procedures. There was no significant association between postoperative complications and the presence of a concurrent procedure (*χ* ^2^ = 0.002, *p* = 0.968; Fisher's exact *p* = 1.000).

**TABLE 4:** Chi‐square tests for proportional variations in surgical success.

Independent variable	Variable classification	Surgical success (% total)	Not surgical success (% total)	*p*
OSA severity	Mild	0 (0.0)	1 (2.3)	0.310
	Moderate	4 (9.3)	1 (2.3)	
	Severe	22 (51.2)	15 (34.9)	
Presence of severe OSA	Not severe	4 (9.3)	2 (4.7)	0.738
	Severe	22 (51.2)	15 (34.9)	
Presence of obesity	Obese	12 (27.9)	5 (11.6)	0.272
	Not obese	14 (32.6)	12 (27.9)	
Presence of syndrome	Syndromic	14 (32.6)	11 (25.6)	0.480
	Non‐syndromic	12 (27.9)	6 (14.0)	
Gender	Male	12 (27.9)	10 (23.3)	**0.039**
	Female	16 (37.2)	5 (11.6)	
Concurrent procedure	Yes	7 (16.3)	4 (9.3)	0.803
	No	19 (44.2)	13 (30.2)	

*Note*: Concurrent procedures were defined as an additional procedure other than epiglottopexy in the same surgical episode. Percent total is calculated from the total number of patients with both pre‐ and postoperative sleep study data (*n* = 43). Bold values represent statistically significant finding.

Abbreviation: OSA, obstructive sleep apnea.

The multinomial binary logistic regression model did not identify significant predictors of surgical success (Table 5). In the multivariate linear regression model, preoperative AHI was the only variable which added statistically significantly to the prediction (*β* = −0.560 ± 0.168, *p* = 0.002; Table 5). Of the multiple univariate logistic regression analyses, only female gender resulted as a statistically significant predictor (Odds Ratio: 3.840, 95% CI: 1.07–14.21, *p* = 0.044; Table SII).

**TABLE 5:** Multivariate regression variable statistics.

Variable	Multinomial logistic regression			Multivariate linear regression	
	Regression coefficient (±SE)	Odds ratio (95% CI)	Significance	Regression coefficient (±SE)	Significance
Gender	−1.392 (0.957)	0.248 (0.04–1.61)	0.905	−18.221 (9.818)	0.072
Age (years)	−0.142 (0.116)	0.868 (0.69–1.09)	0.221	0.495 (1.191)	0.680
Presence of syndrome	1.428 (0.875)	4.17 (0.75–23.2)	0.103	4.240 (8.452)	0.619
Preoperative BMI	0.019 (0.018)	1.02 (0.69–1.09)	0.293	−0.085 (0.180)	0.639
Preoperative AHI	0.011 (0.017)	1.01 (0.98–1.04)	0.525	−0.560 (0.168)	**0.002**
Preoperative O2 nadir	−0.472 (3.951)	0.62 (0.00–1438.4)	0.905	78.703 (39.94)	0.057

*Note*: Bold values represent statistically significant finding.

Abbreviations: AHI, apnea hypopnea index; BMI, body mass index; CI, confidence interval; n/a, not applicable; SE, standard error.

Eleven patients (19.6%) experienced a postoperative complication (Table 6). Six patients (10.7%) bled postoperatively and 5 (8.9%) required operative intervention. Five patients (8.9%) experienced readmission for pain, dehydration, and/or poor oral intake following initial postoperative discharge. Two patients (3.6%) experienced new postoperative oropharyngeal scarring. One patient (1.8%) had dysphagia lasting beyond 30 days postoperatively.

**TABLE 6:** Postoperative complication information.

Complication	Count (% total)
Any complication	11 (19.6)
Bleed	6 (10.7)
OR control	5 (8.9)
Dysphagia	1 (1.8)
Oropharyngeal scar	2 (3.6)
Readmission	5 (8.9)

*Note*: Dysphagia is defined as documented difficulty with oral intake with or without additional means of enteral access persisting beyond 1 month postoperatively. Readmission was any instance of hospital readmission following initial postoperative discharge for pain, dehydration, and/or poor oral intake.

Abbreviation: OR, operating room.

## 4 Discussion

This retrospective, multi‐institutional study presents the largest reported cohort of pediatric patients with OSA who underwent LT using TORS (*n* = 56) [6, 7, 11, 23, 24]. Additionally, this represents the largest cohort in which most patients (73.2%) did not undergo a concurrent upper airway procedure at the time of LT, regardless of surgical modality [25, 26]. Our findings demonstrate that TORS LT is an effective treatment for pediatric OSA. Of the 43 patients with available postoperative polysomnogram data, 26 (60.5%) met our definition of surgical success, defined as a postoperative AHI < 5 or a ≥ 50% reduction from baseline. Across the cohort, there was a mean postoperative decrease in AHI by 14.4 points (*p* = 0.008). This is consistent with existing pediatric TORS LT cohorts, which report AHI reductions ranging between 13.2 and 16.2 points [7, 23]. This was also consistent with other LT modalities, including unipolar diathermy, endoscopic‐assisted coblation, microdebrider dissection, and radiofrequency ablation, with AHI reductions ranging between 7.3 and 18 points [9, 10, 12, 21, 25]. There were additional significant improvements in REM AHI (*p* < 0.001) and O2 nadir (*p* = 0.002). While this investigation was not a direct head‐to‐head comparison, our results compare similarly with published efficacy in other pediatric TORS lingual tonsillectomy and non‐robotic tonsillectomy techniques.

In comparison to the published success rates for pediatric TORS LT and the published success rates for non‐robotic techniques, our results compare favorably and indicate that this novel technique performs at least as well as others.

Reported success rates of LT, regardless of surgical approach, vary widely (17%–71%), though direct comparisons are limited by differences in baseline severity [21, 25]. While standardized criteria such as the Sher criteria exist for adult OSA, no equivalent definition is established for pediatric patients [27]. Most studies investigating pediatric OSA surgery outcomes have used benchmarks of AHI < 5 or AHI < 1, the latter often termed “surgical cure.” [12, 21, 25, 26] However, some investigations do not explicitly report surgical success rates, further complicating comparisons. Our cohort had a mean preoperative AHI of 35.9 ± 30.6, significantly higher than those in previous LT cohorts (range: 8.5–27.1), and higher preoperative AHI has been shown to be an independent predictor of surgical failure [9, 12, 21, 25, 28, 29, 30, 31]. Thus, using a strict AHI cutoff (e.g., AHI < 5) to define surgical success inherently biases results against cohorts with more severe baseline disease. As such, we sought to report raw mean AHI differences in addition to surgical success definitions.

DS is known to predispose patients to OSA and is associated with higher rates of OSA refractory to conventional adenotonsillectomy due to various anatomical and neurological factors [12, 29]. Notably, lingual tonsillar hypertrophy, relative macroglossia, and midface hypoplasia, which increase the relative volume of the tongue within the oropharyngeal cavity, contribute to greater tongue base obstruction [32]. The majority (93.1%) of our syndromic cohort had DS. Of the 25 syndromic patients with both pre‐ and postoperative polysomnograms, 14 patients (56.0%) were considered a surgical success. Moreover, the frequency of complications in the syndromic cohort did not differ significantly from the non‐syndromic cohort (*χ* ^2^ = 0.220, *p* = 0.639). Our findings support the safety and efficacy of TORS LT in patients with DS with persistent OSA and align with recent large‐scale studies showing no increased risk of surgical failure in this population [25].

In our cohort, the presence of obesity (60.7%) was not associated with differences in surgical success or delta AHI, and BMI was not a significant predictor in either univariate or multivariate regression models. This contrasts with other LT studies, which have reported higher failure rates in patients with obesity, although these studies explored non‐TORS surgical methods. Our findings demonstrate the efficacy of TORS LT irrespective of obesity status. As additional large‐scale TORS studies are conducted, our research will serve as a foundational reference to assess whether TORS is more effective than other techniques (e.g., coblator‐assisted) in treating pediatric patients with OSA and obesity.

Female patients in our study (42.9%) demonstrated a higher surgical success rate than males in the univariate chi‐squared (*p* = 0.039) and univariate logistic regression analyses (*p* = 0.444). However, no significant differences were observed in either of the multivariable models for surgical success or overall AHI reduction. Further, no significant differences were noted between genders in preoperative AHI (*p* = 0.550) or the prevalence of severe OSA (*p* = 0.786). Overall, this suggests that gender is likely not an independent predictor of surgical outcomes. Differences in gender‐specific outcomes following LT of any surgical modality have not been reported previously. Trandafir et al. noted a directionally lower surgical failure rate in females following coblator‐assisted LT (19% vs. 37%), though this difference did not reach statistical significance (*p* = 0.07) [25]. Gender‐specific differences in surgical success are not reported following hypoglossal nerve stimulation and are conflicting following adenotonsillectomy [33, 34, 35]. While estrogen and progesterone may contribute to airway tone preservation and tonsillar regression, their clinical impact on LT surgical success remains unproven [36]. The observed difference in success rates based on gender may be due to confounding or limited sample size.

Eleven patients (19.6%) experienced at least 1 complication. The most common complication was postoperative bleeding (6 patients, 10.7%), 5 of whom required operative intervention. Only 1 patient (1.8%) experienced prolonged dysphagia. This patient was a 13‐year‐old female with DS and a preoperative AHI of 100 with florid lingual tonsillar hypertrophy who had an incidental resection of the superior half of the epiglottis during her LT. She had immediate postoperative dysphagia and aspiration. She required percutaneous gastric tube insertion for several months and underwent intensive feeding therapy. The gastric tube was eventually removed, and she resumed a normal oral diet without aspiration.

Two patients (3.6%) experienced postoperative oropharyngeal scarring. One patient had a revision tonsillectomy at the time of the LT, which created a raw surface at the lateral oropharynx and was the likely cause of oropharyngeal scarring. This patient underwent a subsequent z‐plasty to re‐orient the oropharyngeal tissue to avoid any future issues with intubation, although the patient had an excellent surgical result in terms of AHI reduction and cure of his OSA and had no dysphagia. The second patient was a female with a history of DS and was status post heart transplantation. Her lingual tonsil pathology revealed post‐transplant lymphoproliferative disorder with florid follicular hyperplasia, which may have contributed to excessive inflammation of the surgical bed, resulting in undue scarring. Similarly, she had an excellent reduction of her AHI (preoperative 11.9 vs. postoperative 1.4) and no dysphagia symptoms.

Existing literature exploring pediatric TORS LT postoperative complications is limited by the few available publications and small sample sizes. Leonardis et al. reported 2 patients (12.5%) with postoperative bleeds and 4 patients (25%) with poor postoperative pain control in their cohort of 16 patients [6]. Furthermore, Thottam et al. reported 1 (11.1%) postoperative bleed in their cohort of 9 patients [7].

Overall, these findings suggest that TORS LT is a safe and efficient procedure with minimal long‐term complications. Adequate exposure of the tongue base is crucial to successful lingual tonsillectomy surgery. Our group posits that the use of the robot allows for excellent visualization during monopolar cautery dissection at the lingual tonsil/tongue musculature interface, thus achieving a cleaner and more thorough dissection plane compared with other existing transoral techniques—such as laser or bipolar electrocautery, microdebrider‐assisted, or endoscopic‐assisted coblation—which do not define this interface as easily. While head‐to‐head comparative analyses are lacking at this time, we postulate that such advantages likely lead to a potentially larger volume of lingual tonsil resection.

Our study is not without limitations. While our cohort represents the largest reported for TORS LT, the sample size (56 patients) remains relatively small, particularly limiting the statistical power of our regression analyses. Future studies with larger cohorts are needed to enhance statistical robustness. Additionally, the study design was retrospective. Moreover, it is well known that our primary outcome measure, AHI, does not fully capture the heterogeneity of pediatric OSA [37]. Incorporating metrics such as hypoxic burden, total sleep time under varying oxygen saturation thresholds, and behavioral assessments would strengthen the findings. Furthermore, the authors did not perform a pre‐ and postoperative quality‐of‐life analysis, and validated pain metrics were not collected in the immediate postoperative period. This may be valuable in future work to help contextualize the results in this complex patient population where eradication of OSA is not always possible due to a multitude of contributing factors. Finally, we acknowledge that TORS is not universally available at all institutions, incurs significant costs, and necessitates specialized training.

## 5 Conclusion

This study is the largest series to date investigating pediatric TORS LT. The majority of our cohort was considered surgical successes, and the complication rates were comparable to previously published non‐TORS techniques. TORS is shown to be a safe and effective approach for performing LT in select patients, with specific benefits including improved visualization and differentiation between the lingual tonsil/tongue musculature interface. Potential downsides of TORS are the high cost of purchasing the robot and the necessity of specialized training, although as implementation progresses, these downsides can be minimized. Direct head‐to‐head comparisons between existing pediatric LT techniques, along with larger studies incorporating and complementary sleep and quality of life metrics, are needed to guide discussions on the ideal surgical modality.

## Funding

The authors have nothing to report.

## Conflicts of Interest

The authors declare no conflicts of interest.

## Supporting information


**Table SI:** T‐tests or ANOVA for differences in AHI.
**Table SII:** Univariate logistic regression variable statistics.

## Data Availability

The data that support the findings of this study are available from the corresponding author upon reasonable request.
