# Supplementary material for: Transoral Robotic Lingual Tonsillectomy for Pediatric Obstructive Sleep Apnea
Source: Laryngoscope. 2025 Nov 26;136(5):2332–9. doi: 10.1002/lary.70267 (PMC13067219; doi:10.1002/lary.70267)
Supplement: Supplementary file 1 — Table SI: T‐tests or ANOVA for differences in AHI. Table SII: Univariate logistic regression variable statistics. [file LARY-136-2332-s001.docx]

**SUPPLEMENTAL TABLE I.** T-tests or ANOVA for Differences in Delta AHI.

| **Independent variable** | **Variable classification** | **N value** | **Mean delta AHI (± SD)** | **P value** |
| --- | --- | --- | --- | --- |
| OSA severity | Mild | 1 | +8.00 (0) | 0.586 |
|  | Moderate | 5 | -3.40 (2.2) |  |
|  | Severe | 37 | -16.48 (33.8) |  |
| Presence of severe OSA | Not severe | 6 | -1.50 (5.0) | 0.320 |
|  | Severe | 37 | -16.5 (36.0) |  |
| Presence of obesity | Obese | 17 | -11.4 (41.8) | 0.648 |
|  | Not obese | 26 | -16.3 (28.1) |  |
| Presence of syndrome | Syndromic | 25 | -18.9 (30.7) | 0.312 |
|  | Non-syndromic | 18 | -8.18 (37.7) |  |
| Gender | Male | 22 | -7.28 (36.8) | 0.161 |
|  | Female | 21 | -21.8 (29.4) |  |

AHI, apnea hypopnea index; ANOVA, analysis of variance; OSA, obstructive sleep apnea, SD, standard deviation.

**SUPPLEMENTAL TABLE II.** Univariate Logistic Regression Variable Statistics.

| **Variable** | **Exp (B) – odds ratio** | **Significance** |
| --- | --- | --- |
| Gender | 9.23x10^8^ | 0.998 |
| Age (years) | 0.895 | 0.304 |
| Presence of syndrome | 2.821 | 0.200 |
| Presence of obesity | 0.591 | 0.505 |
| BMI | 1.012 | 0.476 |
| Presence of severe OSA | 0.857 | 0.895 |
| AHI | 1.022 | 0.277 |
| O2 nadir | 35.177 | 0.207 |

AHI, apnea hypopnea index; BMI, body mass index; OSA, obstructive sleep apnea.
